# Supplementary material for: Cyclic peptide FXII inhibitor provides safe anticoagulation in a thrombosis model and in artificial lungs
Source: Nat Commun. 2020 Aug 4;11:3890. doi: 10.1038/s41467-020-17648-w (PMC7403315; doi:10.1038/s41467-020-17648-w)
Supplement: Supplementary file 1 — Supplementary Information [file 41467_2020_17648_MOESM1_ESM.pdf]

Cyclic peptide FXII inhibitor provides safe anticoagulation in a thrombosis model and in artificial lungs

Wilbs et al.

Supplementary Figures

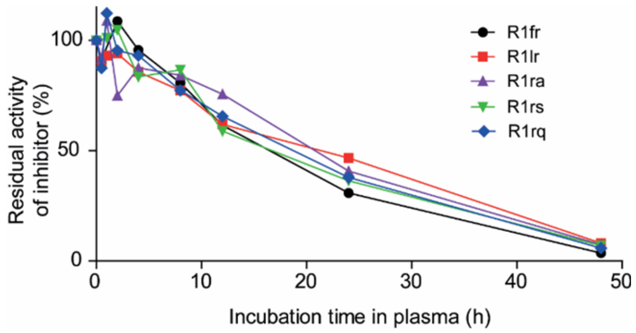

Supplementary Fig. 1. Stability of FXI618 variants carrying two D-amino acids at the N-terminus. The peptides were incubated in human plasma as indicated. Residual inhibitory activity was tested in a FXIIa activity assay using a fluorogenic substrate. Residual inhibition in % was calculated as $(IC_{50,0h}/IC_{50,xh}) \times 100$, wherein $IC_{50,0h}$ is the functional strength of the inhibitor at time point 0 and $IC_{50,xh}$ is the functional strength of inhibitor after one of the different plasma incubation periods.

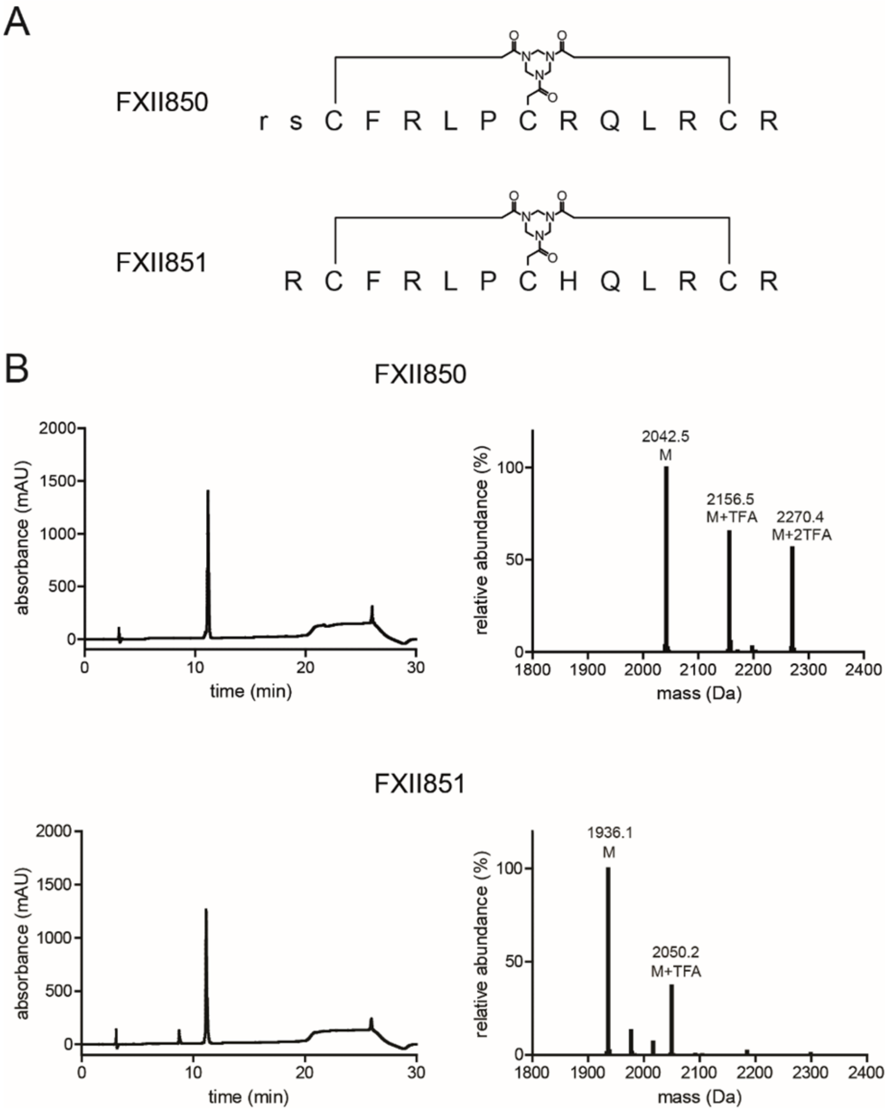

Supplementary Fig. 2. FXIIa inhibitors FXII850 and FXII851. (**A**) Schematic representation of structures. (**B**) Analytical RP-HPLC and MS data. The MS spectra were deconvoluted and show the molecular weight of the uncharged inhibitors.

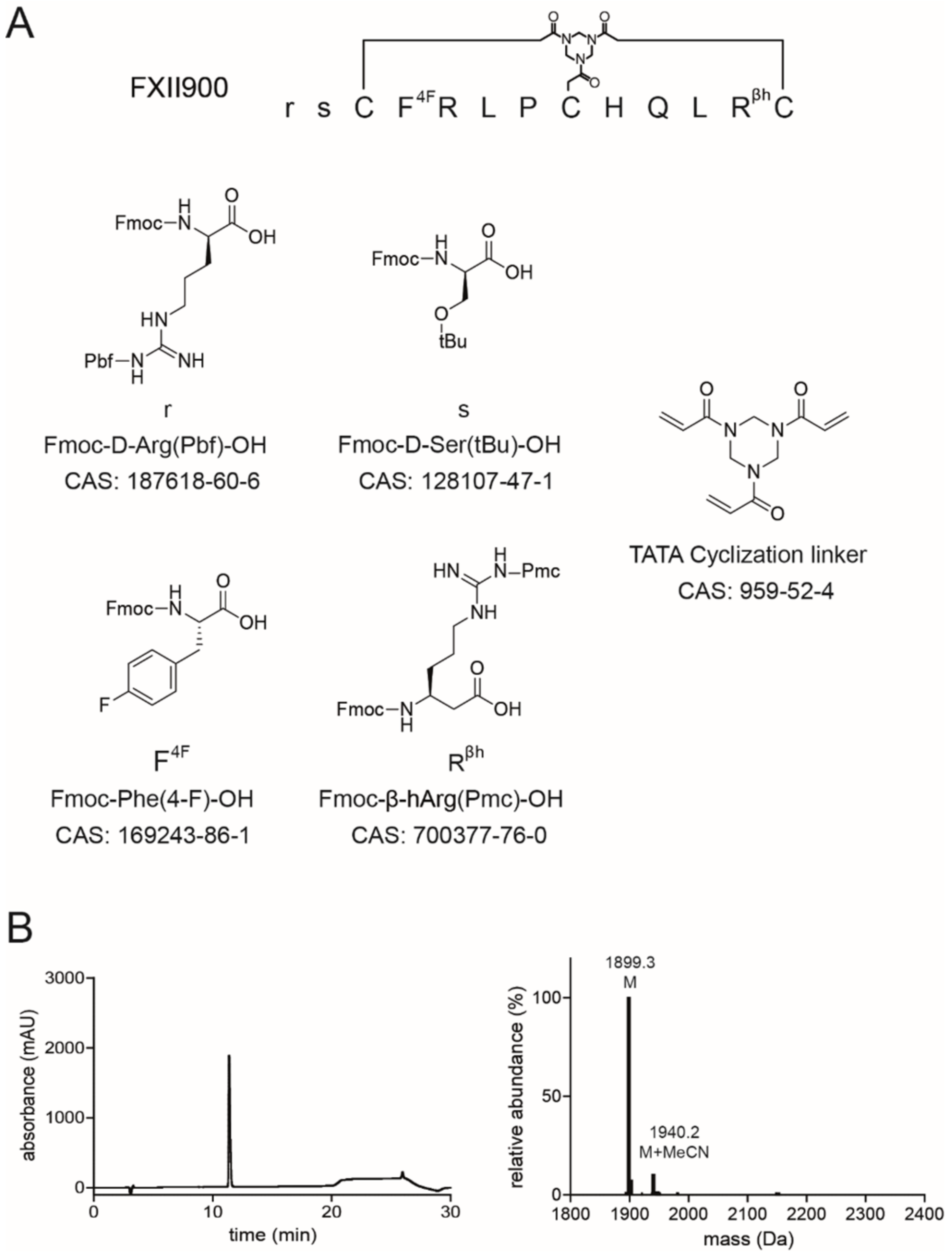

Supplementary Fig. 3. Synthesis of FXII900. (**A**) The structure of FXII900 is shown schematically together with the chemical structures of the unnatural amino acids and the chemical linker. (**B**) RP-HPLC and MS analysis. The MS spectrum was deconvoluted and shows the molecular weight of the uncharged inhibitor.

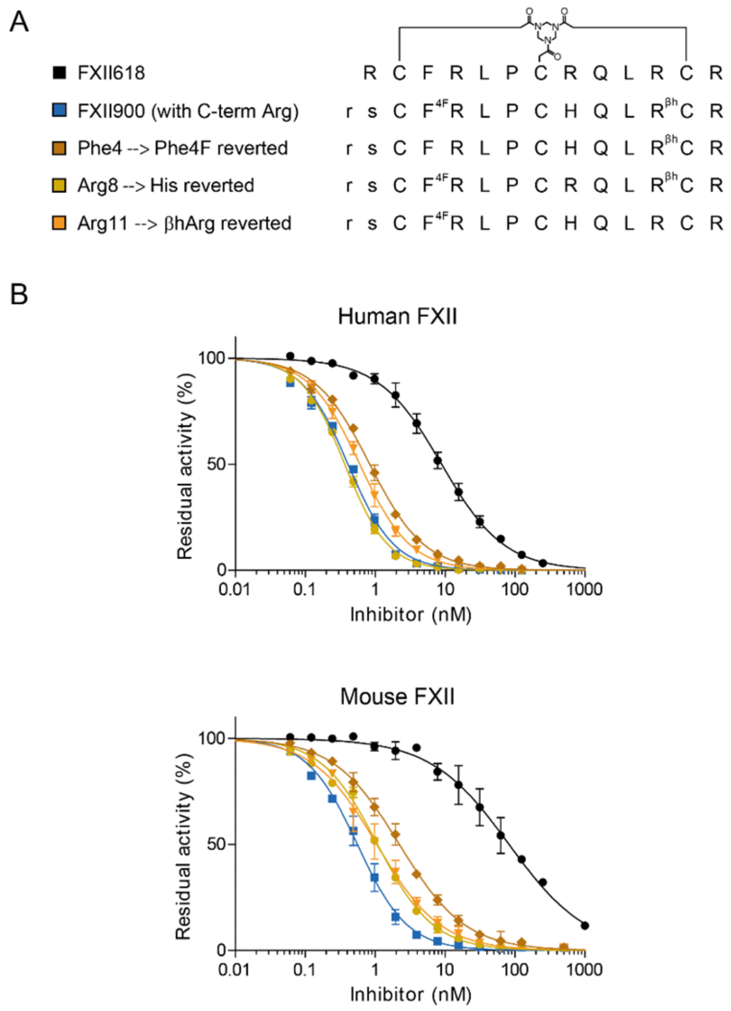

Supplementary Fig. 4. Reversion of beneficial amino acid substitutions in FXII900. The three amino acids Phe4F3, His8, and βhArg11 were individually reverted in FXII900 (containing a C-terminal Arg) to the original residues Phe3, Arg8, and Arg11, in order to assess the contribution of these modifications to the stability of the inhibitor as well as to the inhibition of human and mouse FXIIa. (**A**) The amino acid sequences of the bicyclic peptides are indicated. All peptides contain a C-terminal Arg. (**B**) The residual activity of human and mouse FXIIa at different peptide concentrations was measured in triplicate. Means ± SD are indicated. (**C**) RP-HPLC and MS analysis.

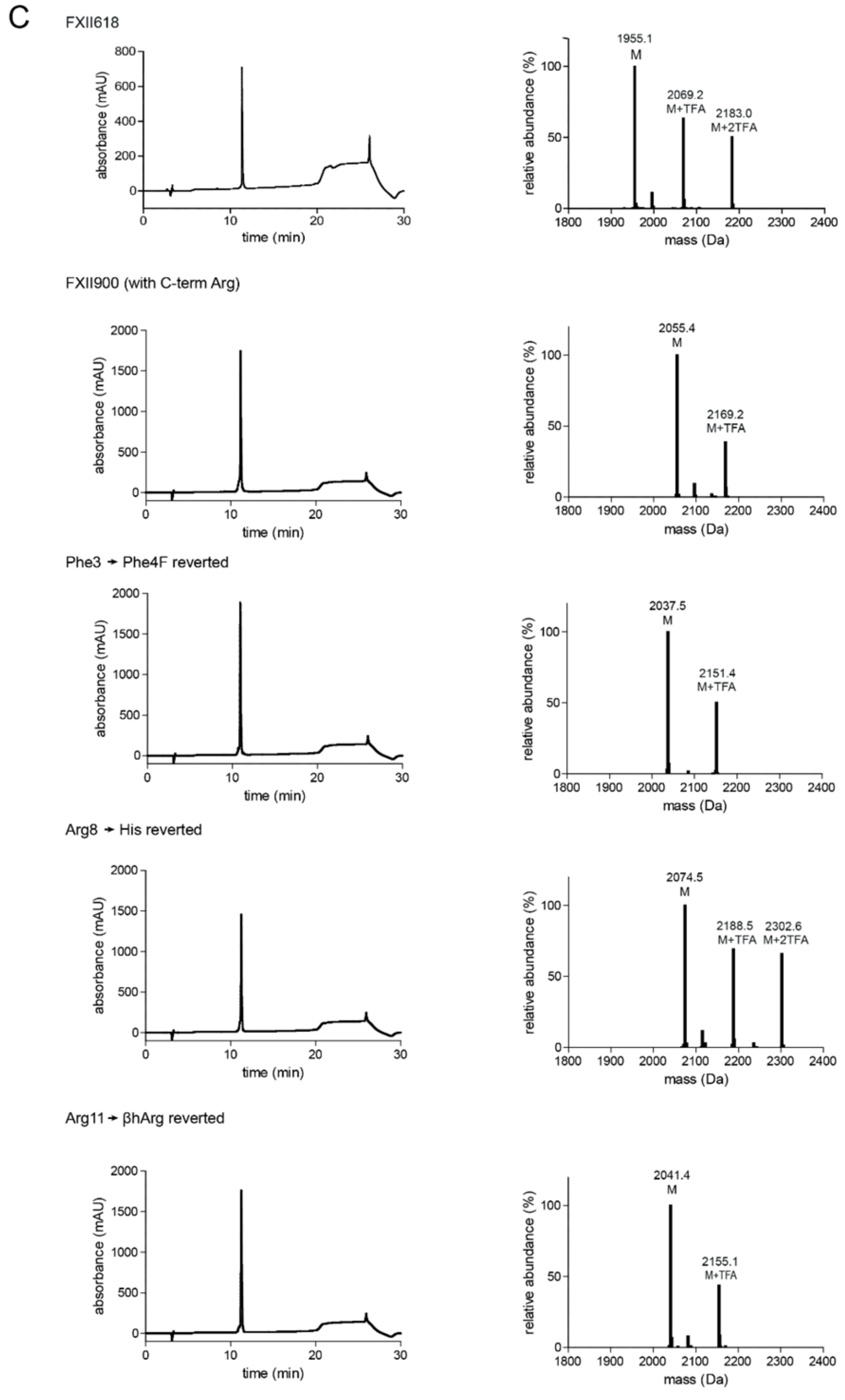

Supplementary Fig. 4. Continued

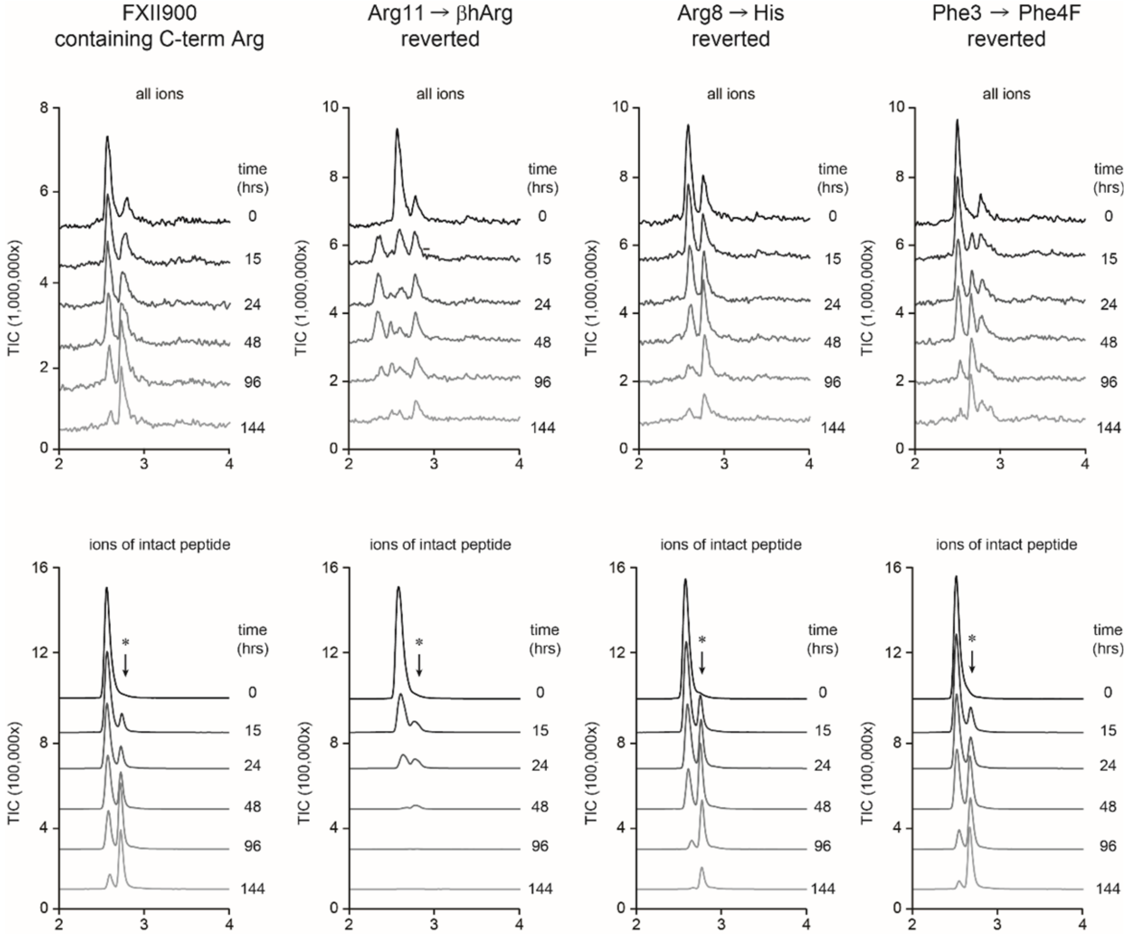

Supplementary Fig. 5. Plasma stability of FXII900 and variants with reverted beneficial mutations. The three amino acids Phe4F3, His8, and βhArg11 were individually reverted in FXII900 containing a C-terminal Arg to the original residues Phe3, Arg8, and Arg11, respectively, and the stability in mouse plasma was assessed. Peptides were incubated at a concentration of 80 µM in mouse plasma at 37°C. After precipitation and removal of plasma proteins, the peptides and their degradation products were analyzed LC-MS. Total ion counts (TIC) are indicated in the top panels. The lower panels show the ion count of the intact peptides. For all peptides, a species was found with an additional mass of one dalton. It is assumed that this mass results from the hydrolysis of the C-terminal amide group.

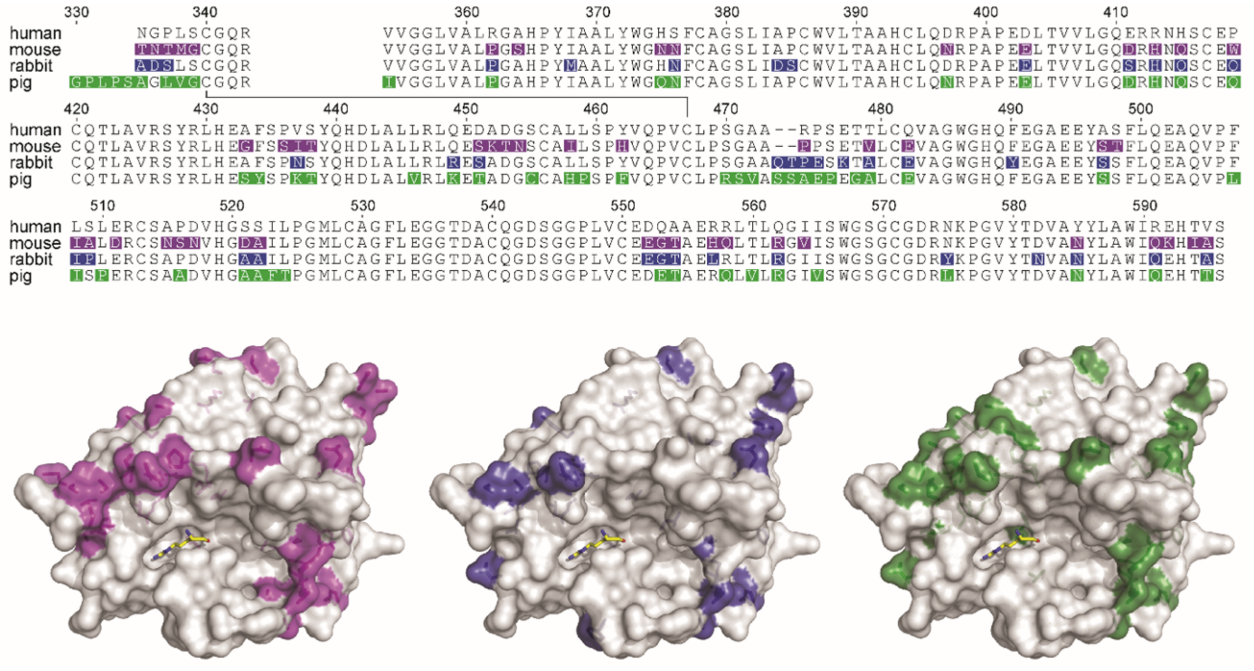

Supplementary Fig. 6. Comparison of human, mouse, rabbit and pig FXII. The amino acid sequences of β-FXIIa of human, mouse, rabbit and pig FXII are aligned. Amino acids that are different compared to the human protein are highlighted in color. The same amino acid differences are shown in the β-FXIIa structure below. The structure is a FXIIa homology model that was generated using the sequence of human FXIIa, the X-ray structure of bovine β-trypsin (PDB entry 1PPE) and an alignment scripts from MODELER[1], and was described before.[2] An arginine residue was placed into the S1 specificity pocket (yellow) to indicate the inhibitor binding site and orientation of the protease.

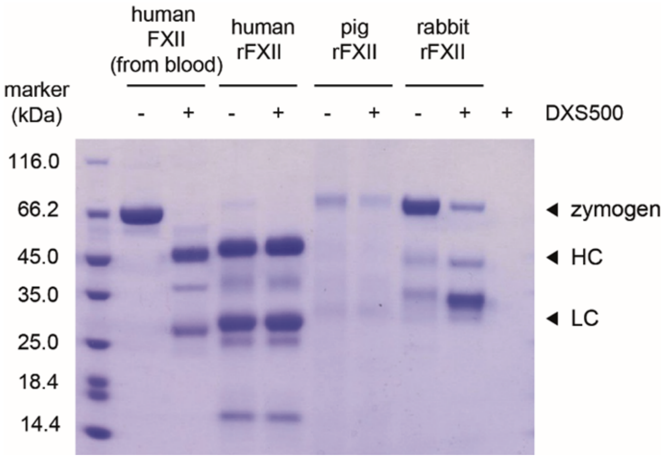

Supplementary Fig. 7. Recombinant expression of human, rabbit and pig FXII. The recombinant proteins were expressed in CHO cells and purified by immobilized metal ion affinity chromatography. Human FXII zymogen derived from blood and recombinant FXII (rFXII) of the different species were analyze by SDS-PAGE under reducing conditions before and after incubation with dextran sulfate (DXS500). Dextran sulfate leads to auto-activation and cleavage of FXII into a heavy chain (HC) and a light chain (LC). The human rFXII was already activated during the expression or purification. The pig rHFXII was not cleaved into HC and LC. An activity assay showed that pig rHFXII treated with dextran sulfate was active, suggesting that it was converted into the active α-FXIIa, but not further processed into HC and LC. The recombinantly expressed human, pig and rabbit FXII were analyzed by SDS-PAGE a second time with similar results.

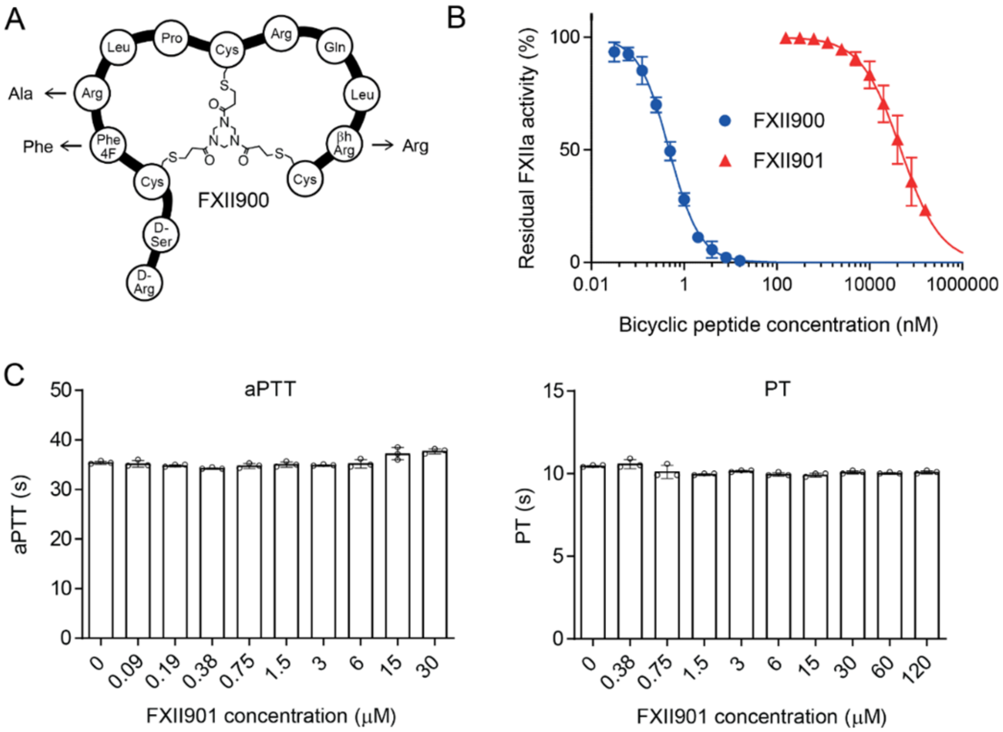

Supplementary Fig. 8. Negative control peptide FXII901. (**A**) The amino acid substitutions made in FXII900 to render it inactive at low micromolar concentrations. (**B**) Inhibition of FXIIa by FXII900 and FXII901. Means ± SD of three measurements are shown. Data of individual measurements are shown as dots. (**C**) Effect of FXII901 on aPTT and PT in human plasma. The coagulation times were measured in triplicate and means ± SD are indicated.

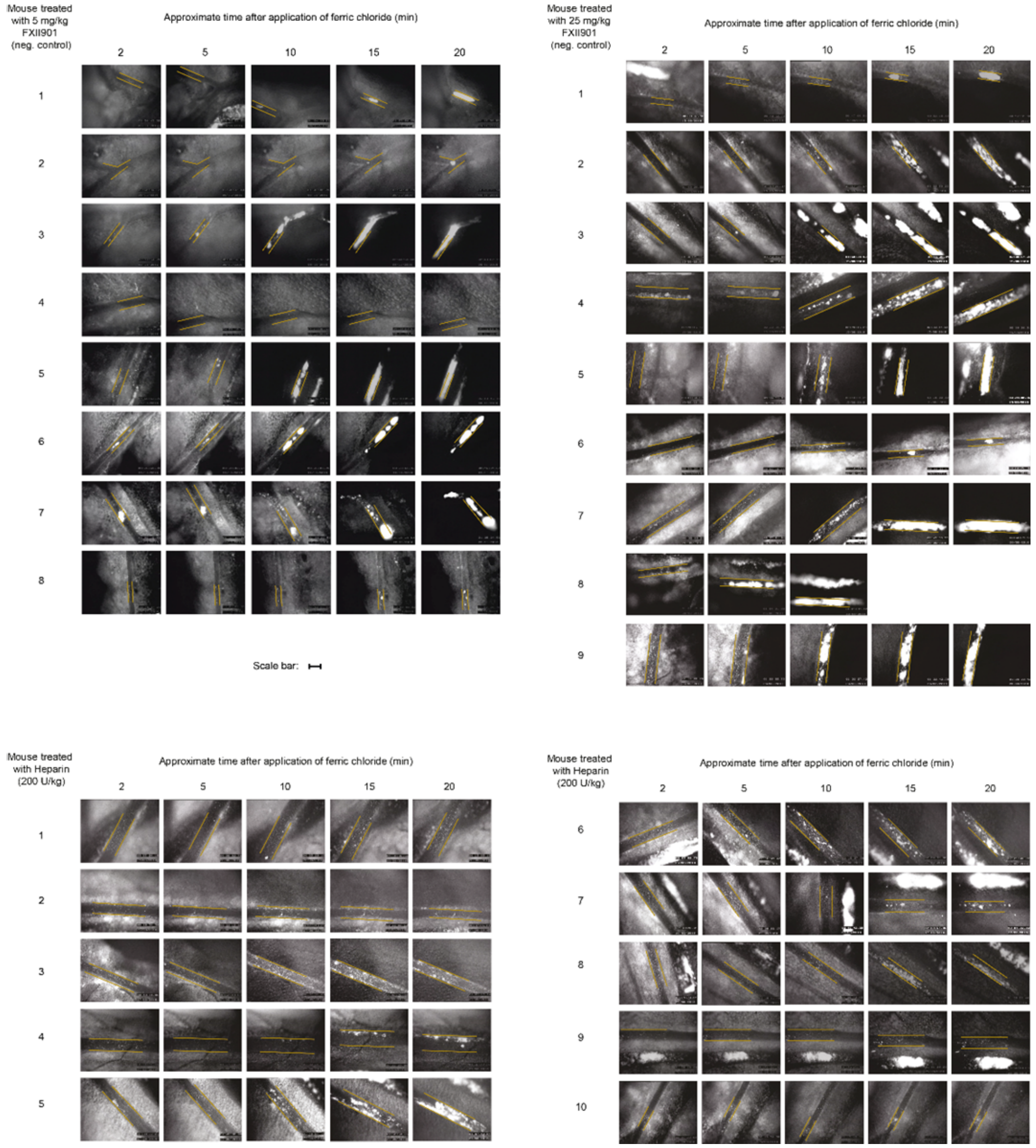

Supplementary Fig. 9. Ferric chloride-induced thrombosis in mice treated with negative control peptide FXII901 or the positive control heparin. Intravital fluorescence microscopy images of mesenteric arterioles in which thrombosis was induced by topical application of $FeCl_3$ (7.5%, 1 min). Platelets were fluorescently labeled with Rhodamine 6G for visualization. Vessel walls at the $FeCl_3$ application site are indicated with yellow markers. The mesenteric vessels were imaged at additional time points and similar results were obtained for similar time points. Scale bar: 200 μm.

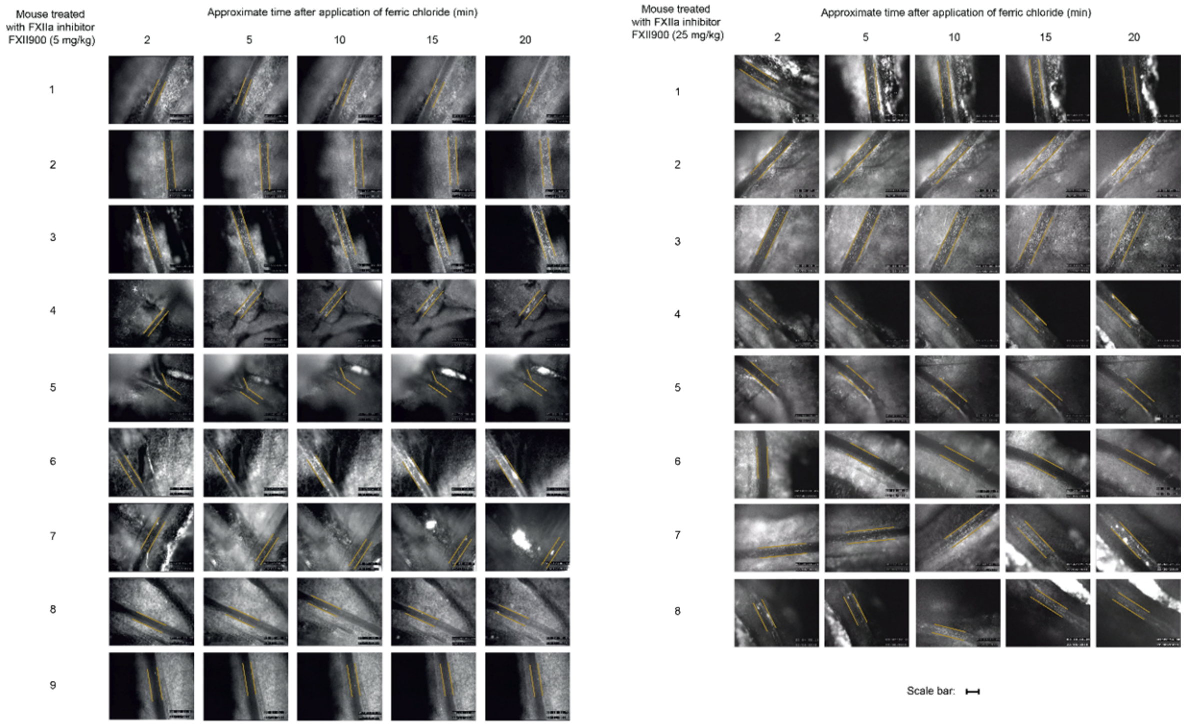

Supplementary Fig. 10. Ferric chloride-induced thrombosis in mice treated with FXIIa inhibitor FXII900. Intravital fluorescence microscopy images of mesenteric arterioles in which thrombosis was induced by topical application of $FeCl_3$ (7.5%, 1 min). Platelets were fluorescently labeled with Rhodamine 6G for visualization. Vessel walls at $FeCl_3$ application site are indicated with yellow markers. The mesenteric vessels were imaged at additional time points and similar results were obtained for similar time points. Scale bar: 200 μm.

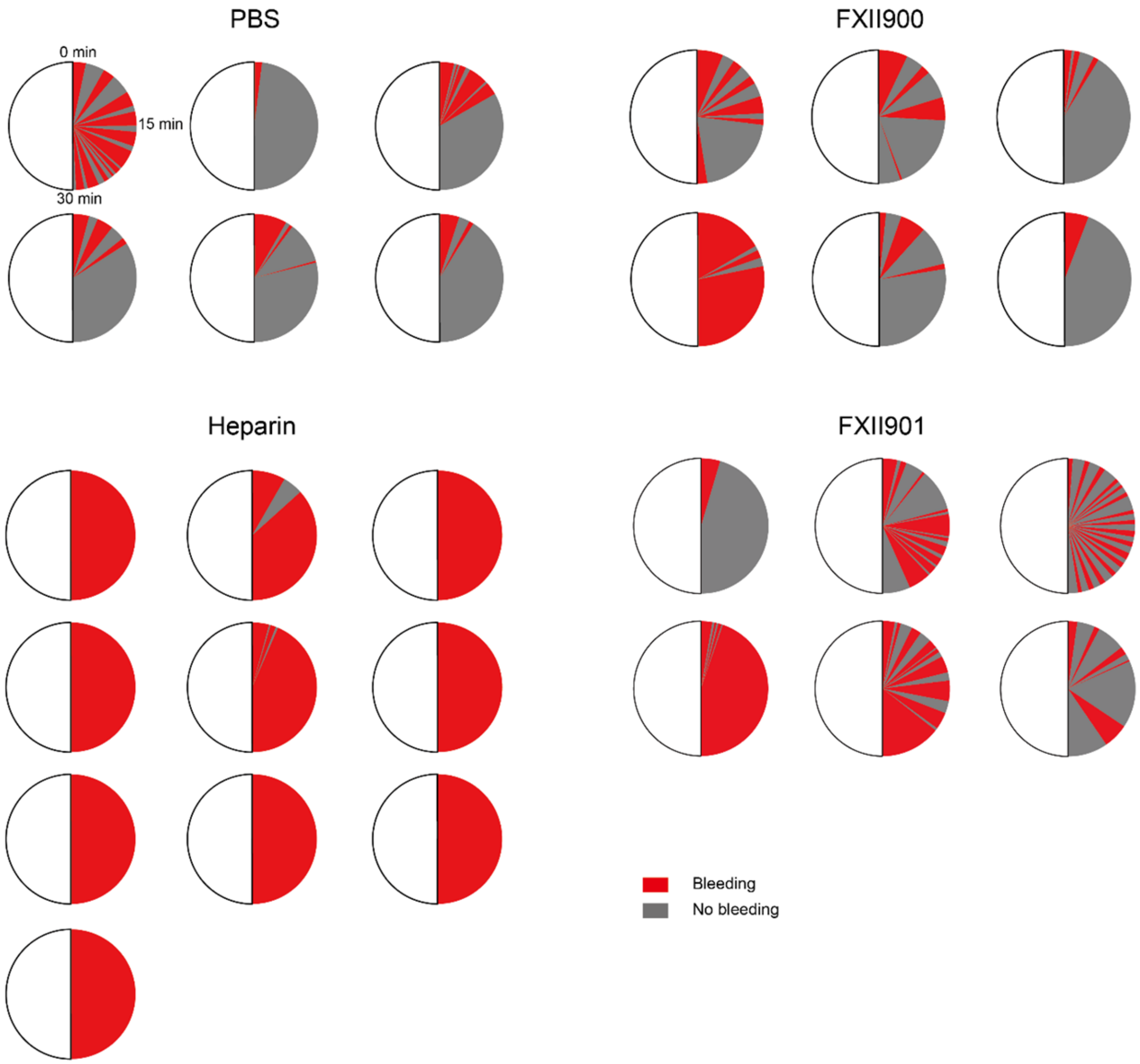

Supplementary Fig. 11. Bleeding in mice injected with PBS, heparin, FXII900 or FXII901. The graphs indicate the time periods at which mice with 2 mm tail transections were bleeding. Mice were treated 5 minutes before clipping the tail tips with vehicle (PBS, IV, n = 6), heparin (200 IU kg^{-1}, IV, n = 10), FXII900 (25 mg kg^{-1}, SC, n = 6) or FXII901 (25 mg kg^{-1}, SC, n = 6)

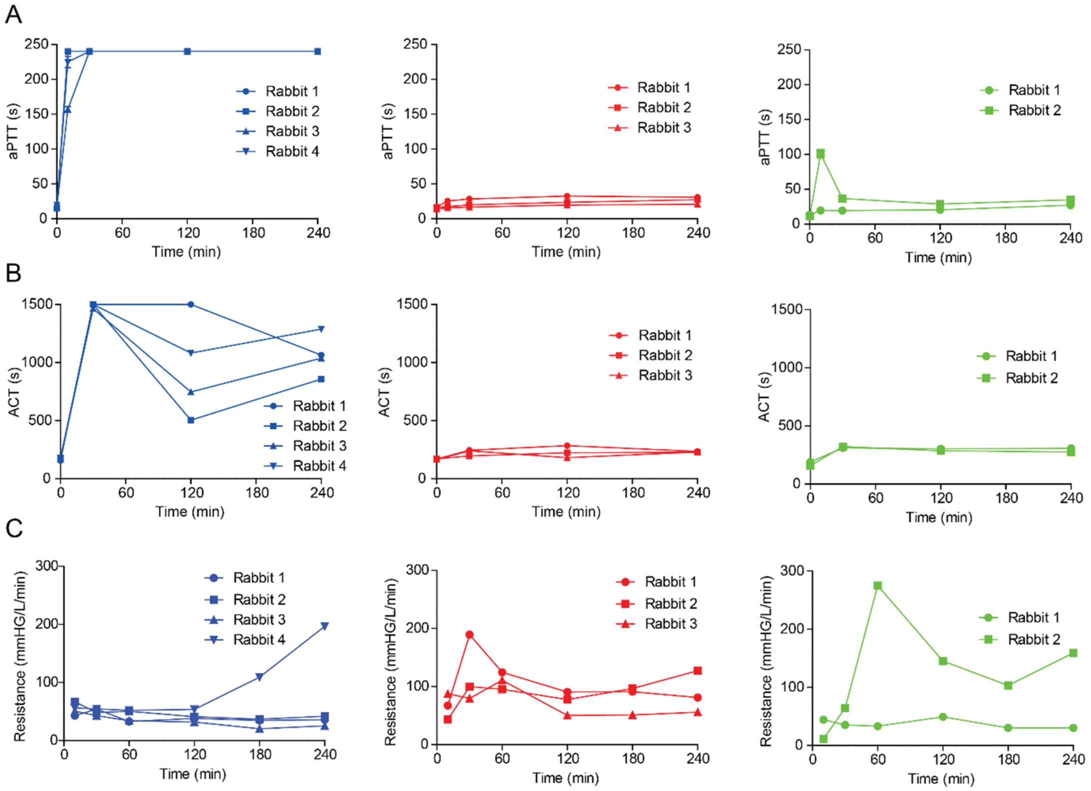

Supplementary Fig. 12. Inhibition of FXIIa in artificial lung rabbit model: data for individual rabbits. Rabbits connected veno-venous to an artificial lung system for four hours were injected with FXII900 as a bolus (2 mg kg^{-1}) before start of the extracorporeal circulation and as a constant infusion (0.075 mg kg^{-1} min^{-1}) over the full time course of the experiment (n = 4; data shown in blue), or were not treated (n = 3, data shown in red), or infused initially with one IU per min heparin wherein the drip rate was adjusted to maintain ACT values between 220 and 300 s (n = 2, data shown in green). Mean values ± SD for the three groups are shown in Fig. 5. Values indicated for the time point 0 refer to measurements made before connection of the artificial lung. (**A**) aPTT. Coagulation times of 240 s indicate that plasma did not coagulate for 240 s, at which point the assay was stopped. (**B**) ACT. Clotting times of 1500 s indicate that blood did not coagulate for 1500 s, at which point the assay was stopped. (**C**) Resistance calculated based on pressure at the inlet and outlet of the device and the flow rate. (**D**) Bleeding

time measured by incision-provoked injuries at the ears. (**E**) Platelet count. The sample of rabbit 1 was lost. (**F**) Volume of blood clots indicated in % of volume of the artificial lungs.

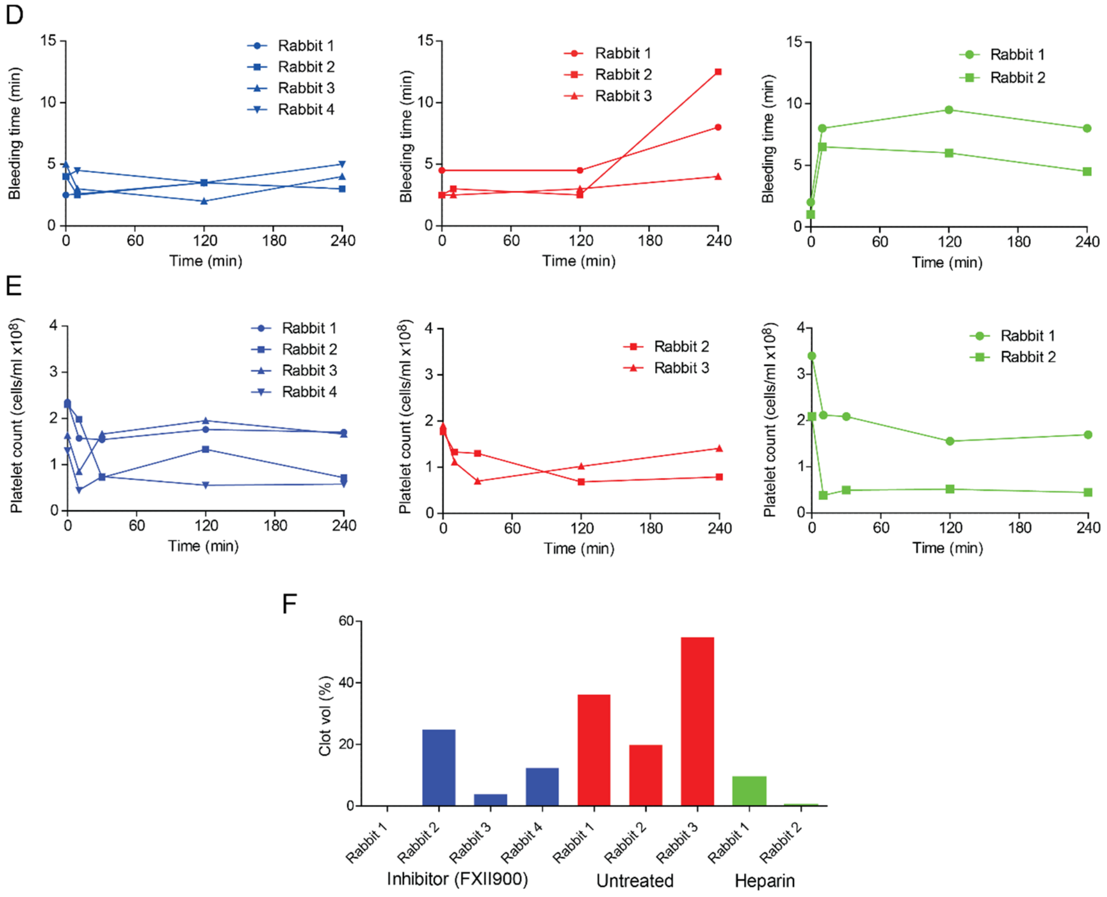

Fig. S12. Continued

Supplementary Tables

Supplementary Table 1. K_i values of coumarin-labeled FXII618 modified at Arg1 or Arg8. All peptides were synthesized with the additional amino acid β-(7-methoxy-coumarin-4-yl)-Ala-OH at the C-terminus for accurate concentration determination. This approach enabled the determination of K_i values for the crude bicyclic peptide. For comparison, the crude bicyclic peptide FXII618 modified with the coumarin amino acid inhibited human and mouse FXIIa with K_i values of 20 ± 3 nM and 230 ± 30 nM, respectively. The K_i values of the crude peptides carrying the coumarin amino acid are around two-fold higher than those of purified peptides without coumarin. If the first measurement showed a K_i below 300 nM, the measurement was repeated and average values are indicated for these peptides. For peptides measured in triplicate, SDs are indicated.

**Modification in Arg1**			**Modification in Arg8**		
**Altered amino acid**	**K_i human FXIIa (nM)**	**K_i mouse FXIIa (nM)**	**Altered amino acid**	**K_i human FXIIa (nM)**	**K_i mouse FXIIa (nM)**
R1ar	32	350	R8A	20.5	230
R1sr	59	750	R8S	44.0	360
R1fr	22	190	R8F	103	900
R1lr	16	131	R8L	44.3	490
R1qr	32	250	R8P	88.8	440
R1ra	12.4	88 ± 4	R8K	24.9	250
R1rs	10.9	85 ± 14	R8H	13.6	88
R1rf	36.3	220	R8E	41.3	730
R1rl	83	440	R8Q	38.3	330
R1rq	21	160	R8N	51	330

Supplementary Table 2. Overview of FXII618 variants and their K_i values. K_i values were measured two (FXII850, FXII851), three (FXII618, FXII700, FXII800) or five times (FXII900). Mean values are shown. Standard deviations are indicated if values were measured at least three times.

**Inhibitor name**	**Modification to FXII618**	**K_i human FXII (nM)**	**K_i mouse FXII (nM)**	**Reference**
FXII618	-	8.1 ± 0.7	86 ± 9	Baeriswyl, V., *et al.*, ACS Chem. Biol., 2015
FXII700	Arg11→βhArg	1.5 ± 0.1	N.D.	Wilbs, J., *et al.*, ChemBioChem, 2016
FXII800	Phe3→Phe4F	0.84 ± 0.03	N.D.	Middendorp, S., *et al.*, J. Med. Chem., 2017
FXII850	Arg1→D-Arg-D-Ser	5	35	-
FXII851	Arg8→His	6.5	36	-
FXII900	Arg11→βhArg, Phe3→Phe4F, Arg1→D-Arg-D-Ser, Arg8→His, deletion of Arg13	0.37 ± 0.04	0.45 ± 0.11	-

Supplementary Table 3. Specificity of FXII900. The indicated values are averages of two (most proteases) or three measurements (plasmin, APC). Mean values are shown. Standard deviations are indicated if values were measured three times.

**Protease**	**K_i (nM)**	**% residual activity at 40 µM inhibitor**
FXIa	> 40,000	95.1
Plasmin	> 40,000	86 ± 5
Trypsin	1460	9.2
uPA	> 40,000	98.7
tPA	> 40,000	97
Thrombin	> 40,000	95
Plasma Kallikrein	> 40,000	72
FXa	> 40,000	97.1
FVIIa	> 40,000	97.1
APC	> 40,000	98.3 ± 2.3

Supplementary Table 4. Reversion of beneficial mutations. The indicated values are averages of at least three measurements. Mean ± SD are indicated.

**Peptide**	**K_i human FXII (nM)**	**K_i mouse FXII (nM)**
FXII618	8.1 ± 0.7	86 ± 9
FXII900	0.37 ± 0.04	0.45 ± 0.11
FXII900 (with C-term Arg)	0.34 ± 0.03	0.50 ± 0.15
Phe3→Phe4F reverted	0.70 ± 0.06	1.9 ± 0.5
Arg8→His reverted	0.31 ± 0.02	0.91 ± 0.04
Arg11→βhArg reverted	0.51 ± 0.10	0.9 ± 0.4

Supplementary Table 5. Pharmacokinetic parameters in rabbits. FXII900 was administrated intravenously (n = 3, 3.7 mg kg^{-1}) and the concentration was quantified in duplicate (rabbit 1) or triplicate (rabbit 2 and 3) by LC-MS.

**Parameter**	**Value**
$t_{1/2}$ β (min)	12 ± 2
CL (ml kg^{-1} min^{-1})	16.1 ± 1.0
V_D (ml kg^{-1})	280 ± 50

Supplementary Table 6. Pharmacokinetic parameters in pigs. FXII900 was administrated intravenously (n = 3, 4 mg kg^{-1}) and the concentration was quantified by LC-MS in duplicate for each pig.

Parameter	Value
$t_{1/2}$ β (min)	36 ± 5
CL (ml kg^{-1} min^{-1})	11.8 ± 1.8
V_D (ml kg^{-1})	610 ± 140

Supplementary Table 7. Time to clot formation and full occlusion in ferric chloride-induced thrombosis mouse model. Time points at which clot formation and blood vessel occlusion were observed.

	Control (5 mg ml^{-1})		Control (25 mg ml^{-1})		Inhibitor (5 mg ml^{-1})		Inhibitor (25 mg ml^{-1})		Heparin (200 U kg^{-1})	
Mouse ID	Clot (min)	Occlusion (min)	Clot (min)	Occlusion (min)	Clot (min)	Occlusion (min)	Clot (min)	Occlusion (min)	Clot (min)	Occlusion (min)
1	14	18	12	15	-	-	-	-	-	-
2	17	-	10	-	-	-	-	-	-	-
3	6	9	3	10	23	-	-	-	15	-
4	-	-	2	19	-	-	19	-	15	19
5	6	9	6	12	-	-	-	-	10	-
6	7	9	10	15	21	-	-	-	10	-
7	2	10	10	13	16	-	18	-	15	-
8	15	-	3	7	-	-	-	-	13	-
9			6	15	-	-			-	-
10									-	-

Supplementary Methods

Peptide Synthesis

Solid-phase peptide synthesis was performed on an Advanced ChemTech 348 Ω peptide synthesizer (AAPPTec) using standard Fmoc procedures. Rink Amide AM resin was used as solid support and dimethylformamide (DMF) as solvent. Peptide variants were synthesized at a 0.03 mmol scale to obtain around 10–20 mg of pure peptide. Peptide couplings were performed twice for each natural amino acid by reacting amino acid (4 eq., 600 µl of 0.2 M in DMF), HBTU/HOBt (4 eq. each, 267 µl of 0.45 M in DMF), and DIPEA (6 eq., 360 µl of 0.5 M in DMF) at RT for 30 minutes at 400 rpm. The coupling of unnatural amino acids (2 eq., 600 µl of 0.1 M in DMF) was performed once using HATU (2 eq., 650 µl of 0.1 M in DMF) and DIPEA (4 eq., 250 µl of 0.5 M in DMF). The resin was washed four times with DMF after the coupling reaction. The N-terminal Fmoc protecting group was removed with piperidine (20% [v/v]) in DMF (RT, 2 × 5 min, 400 rpm). The resin was washed five times with DMF after Fmoc removal. The peptides were synthesized from the C-terminal end to the N-terminus by first coupling a glycine to the resin followed by the coumarin-containing amino acid Fmoc-β-(7-methoxy-coumarin-4-yl)-Ala-OH (Bachem). These two coupling steps as well as HATU couplings were followed by an acetylation capping step using acetic anhydride (1 ml of 10% [v/v] in DMF) and DIPEA (1 ml of 15% [v/v] in DMF) at RT for 30 minutes at 400 rpm. Fmoc-L-α-amino acids, HBTU, HOBt, and Rink Amide AM resin were purchased from GL Biochem. Non-canonical Fmoc-amino acids were purchased from Bachem, Chem-Impex, PolyPeptide, and TCI. Peptides were cleaved from the solid support and protecting groups were removed by incubation in 5 ml cleavage cocktail (90% TFA, 2.5% ethane-1,2-dithiol, 2.5% phenol, 2.5% thioanisole, 2.5% H_2O) for two hours with shaking. The resin was removed by vacuum filtration, and the peptides were precipitated with ice-cold diethyl ether (50 ml), incubated for 30 minutes at -20°C, and pelleted by centrifugation (2700 g, 5 minutes). The diethyl ether was discarded, the precipitate washed twice with diethyl ether, and the remaining solvent evaporated at RT. The linear peptide of FXII900 was synthesized at a one gram-scale by GL Biochem.

Peptides cyclization and purification

Crude peptide at a concentration of 1 mM was reacted with 1.2 mM of TATA in 70% NH_4HCO_3 buffer (60 mM, pH 8.0) and 30% MeCN for one hour at 30°C. The peptide and TATA were prepared and mixed using the solvents, concentrations, and volumes exemplified for the cyclization of ~ 50 mg crude peptide. To 6 ml of 3.5 mM peptide in MeCN/H_2O (1:2), 2.7 ml of 10 mM TATA in MeCN and 2 ml of MeCN were added and the reaction started by the addition of 12 ml NH_4HCO_3 buffer (60 mM, pH 8.0). The cyclized peptides synthesized on a 50 mg scale were purified by reversed-phase HPLC (Waters Prep LC 4000 system) using a preparative C18 RP column (Vydac C18 TP1022 column, 250 × 22 mm, 10 µm, 300 Å) and a linear gradient of 15–28% solvent B (MeCN, 0.1% [v/v)] TFA) in solvent A (H_2O, 0.1% [v/v] TFA) in 19 minutes at a flow of 20 ml/min. Absorbance was detected at 220 nm. The cyclized peptides were injected directly as the reaction mixture or after lyophilization of the reaction by dissolving the peptide in 10 ml of H_2O containing 10% (v/v) MeCN and 0.1% (v/v) TFA. Fractions containing the desired peptide were lyophilized.

HPLC and mass spectrometric analysis

The molecular mass of the purified peptides was determined on a single quadrupole mass spectrometer in positive ion mode using electrospray ionization (LCMS-2020, Shimadzu). The software LabSolutions (version 5) was used to analyze the mass spectra. The purity of the peptides was determined by analytical RP-HPLC (Agilent 1260 HPLC system) using an analytical C18 column (Agilent Zorbax 300SB-C18, 4.6 mm × 250 mm, 5 µm) and a linear gradient of 0–50% solvent B (MeCN, 0.1% [v/v] TFA) in solvent A (H_2O, 5% [v/v] MeCN, 0.1% [v/v] TFA) in 15 minutes at a flow of 1 ml min^{-1}. Absorbance was detected at 220 nm.

Peptide concentration determination

Lyophilized peptide was weighed and dissolved in water to obtain peptide stocks of 1 mM. The concentrations of crude peptides containing the coumarin amino acid were determined by measuring the absorption at 325 nm (ε = 12000 M^{-1} cm^{-1}). The peptides were typically diluted several fold with water to measure the concentration in an absorption range of 0.1–1.

Protease inhibition assays

The inhibitory activity of peptides was determined by measuring the residual protease activity with a fluorogenic substrate. The assay was performed in buffer containing 10 mM Tris-Cl, pH 7.4, 150 mM NaCl, 10 mM $MgCl_2$, 1 mM $CaCl_2$, 0.1% (w/v) BSA, 0.01% (v/v) Triton-X100, and 1% (v/v) DMSO in a volume of 150 µl. FXIIa activity was measured with the fluorogenic substrate Boc-Gln-Gly-Arg-AMC (Bachem) at a final concentration of 50 µM. Mouse α-FXIIa (Molecular Innovations cat # MFXIIA) and human β-FXIIa (Molecular Innovations cat # HFXIIAB) were used at final concentrations of 0.4 nM. The reaction was pipetted as follows: 50 µl of bicyclic peptide in assay buffer were added to 50 µl of FXIIa in assay buffer and incubated for 10 minutes at 25°C before 50 µl of 150 µM fluorogenic substrate in assay buffer were added to measure the residual protease activity with an Infinite M200Pro fluorescence plate reader (excitation at 368 nm, emission at 467 nm; Tecan) for a period of 30 minutes with a read every minute at 25°C. The Tecan software i-control (version 1.1) was used for data analysis. Stock solutions of 1 mM bicyclic peptide were prepared by either dissolving the unpurified cyclization reaction with water or by dissolving purified and lyophilized peptide in water. The bicyclic peptides were serially diluted two-fold using assay buffer. Sigmoidal curves were fitted to the data using the following dose response equation (1), wherein x = peptide concentration, y = % protease activity, and p = Hill slope. IC_{50} values were derived from the fitted curve.

$$(1) \qquad y = \frac{100}{1+10^{(\log IC50 - x)p}}$$

The inhibition constants (K_i) were calculated according to the equation of Cheng and Prusoff, $K_i = IC_{50}/(1+([S]_0/K_m)$, wherein IC_{50} is the functional strength of the inhibitor, $[S]_0$ is the total substrate concentration, and K_m is the Michaelis-Menten constant. A K_m of 260 µM was used for the substrate Boc-Gln-Gly-Arg-AMC.

For testing the specificity of the bicyclic peptides, the following final concentrations of human serine proteases were used: tPA (Molecular Innovations) 7.5 nM, uPA (Molecular Innovations) 1.5 nM, factor XIa (Innovative Research) 6 nM, plasma kallikrein (Innovative Research) 0.25 nM, thrombin (Molecular Innovations) 1 nM, plasmin (Molecular Innovations) 2.5 nM, trypsin (Molecular Innovations) 0.05 nM, factor VIIa (Haematologic Technologies) 50 nM, factor Xa

(Haematologic Technologies) 6 nM, and activated protein C (APC) 5 nM. Two-fold dilutions of the peptide were prepared that ranged from 0.04 to 40 μM. The following fluorogenic substrates were used at a final concentration of 50 μM: Z-Phe-Arg-AMC (Bachem) for plasma kallikrein; Boc-Phe-Ser-Arg-AMC (Bachem) for factor XIa; Z-Gly-Gly-Arg-AMC (Bachem) for tPA, uPA, thrombin, and trypsin; H-D-Val-Leu-Lys-AMC (Bachem) for plasmin; and D-Phe-Pro-Arg-ANSNH-C_4H_9 (Haematologic Technologies) for FVIIa and FXa. For APC, the chromogenic substrate pyroGlu-Pro-Arg-pNA (S2366) was used at a concentration of 400 μM.

DNA sequences

DNA regions that were inserted into a vector for the expression of human, rabbit and pig FXII were verified by Sanger sequencing. The various regions are highlighted using the following color code:

Cleavage sites of *Nhe*I and *Hind*III (black, underlined), BM40 signal sequence (red), gene of FXII (blue), GSGS linker (black), His tag (green).

Human FXII

ATGAGGGCCTGGATCTTCTTTCTCCTTTGCCTGGCCGGGAGGGCTCTGGCAGCTAGCATACCGCCGTGGGAGGCTCCAAAAGAACA
CAAGTACAAGGCTGAAGAGCACACCGTAGTTCTTACGGTAACAGGAGAACCCTGCCATTTTCCATTCCAATATCACCGACAACTCT
ACCACAAGTGCACGCACAAAGGTCGGCCAGGACCGCAGCCGTGGTGTGCAACGACGCCTAACTTCGACCAGGATCAAAGGTGGG
GGTACTGCTTGGAACCAAAAAAAGTGAAAGATCATTGTTCCAAGCATTCACCCTGTCAAAAAGGTGGCACATGTGTGAACATGCC
CAGCGGCCCCCACTGCCTCTGCCCTCAACATTTGACAGGTAACCATTGTCAGAAAGAGAAATGTTTTGAACCCCAATTGTTGAGAT
TTTTTCATAAAAACGAAATCTGGTATCGGACCGAACAGGCCGCGGTGGCGAGGTGTCAATGCAAGGGGCCCGACGCACACTGTCA
GCGACTCGCGAGTCAGGCATGCAGAACCAACCCTTGCCTCCACGGTGGTAGATGTTTGGAAGTAGAGGGACATCGACTTTGTCATT
GTCCTGTTGGCTATACAGGTGCCTTCTGTGATGTCGATACCAAAGCCTCCTGCTATGACGGTCGGGGTCTTAGTTATCGGGGGCTTG
CGCGAACAACCCTCTCTGGTGCTCCCTGTCAGCCCTGGGCATCCGAAGCCACATATCGGAACGTTACTGCGGAGCAGGCCCGGAAT
TGGGGTTTGGGCGGACACGCCTTTTGCAGAAATCCCGATAACGATATTAGACCCTGGTGTTTCGTTCTCAATAGAGATAGGCTCTC
CTGGGAGTACTGCGATCTTGCGCAGTGTCAGACCCCCACGCAAGCCGCCCCTCCCACACCGGTCAGCCCGAGGTTGCACGTTCCTC
TTATGCCTGCGCAACCTGCTCCCCCTAAACCTCAGCCGACCACACGAACTCCACCTCAATCCCAAACTCCAGGTGCACTTCCGGCC
AAGCGCGAGCAGCCTCCAAGTCTTACCCGGAATGGACCCCTTTCATGTGGACAAAGATTGAGAAAATCCCTTTCAAGTATGACAC
GCGTGGTGGGAGGGCTTGTGGCCCTGCGAGGAGCACACCCGTATATAGCGGCACTCTATTGGGGCCATTCATTTTGCGCCGGGTCT
CTGATTGCACCCTGTTGGGTGCTCACCGCGGCCCATTGTCTCCAGGACCGGCCTGCCCCGGAGGACCTGACTGTTGTCTTGGGGCA
GGAAAGGCGCAATCATAGCTGCGAACCATGTCAAACATTGGCAGTTAGAAGCTACCGGCTGCACGAGGCGTTTTCACCCGTGTCTT
ATCAACACGATTTGGCGTTGCTCCGGCTGCAAGAGGACGCTGATGGATCTTGCGCACTCCTCTCACCATACGTGCAGCCCGTGTGC
CTTCCGTCTGGCGCGGCCCGACCGAGCGAAACTACCCTGTGTCAAGTGGCCGGTTGGGGGCATCAGTTCGAGGGGGCGGAGGAGT
ATGCATCCTTCCTCCAGGAAGCCCAAGTCCCTTTCCTTTCTCTCGAAAGATGCTCCGCCCCAGATGTACACGGATCAAGTATTCTTC
CGGGAATGCTTTGTGCGGGGTTTTTGGAAGGCGGCACTGATGCGTGTCAAGGGGACAGCGGGGGACCTCTTGTGTGCGAAGATCA
AGCAGCGGAGAGAAGGTTGACGTTGCAGGGCATTATTAGTTGGGGGAGCGGCTGTGGAGACCGAAATAAGCCGGGCGTCTATACC
GACGTAGCATATTACCTGGCCTGGATACGAGAGCACACGGTGTCCGGCAGTGGCTCCCACCATCATCACCACCACTAAGCTT

Rabbit FXII

ATGAGGGCCTGGATCTTCTTTCTCCTTTGCCTGGCCGGGAGGGCTCTGGCAGCTAGCATCCCCCCCTGGAAGGTACCAAAGGGTCA
TAAACAACGGGCAGACGTGCACATGATGGTTCTTACTGTTACAGGGGAACCCTGTCACTTTCCGTTCCAATATCATCGGCAGTTGT
ATCATACCTGTATACATAAAGGGCGGCCCGGACCGCGCCCATGGTGTGCGACTACTCCAAACTTCGATCGAGACCAGCAATGGGC
ACATTGTCTTGAACATAGGGACGTTAAAGATCATTGTAGTAAGCACTCCCCTTGCCACAAGGGAGGGACCTGTGTAAACACACCTA
GAGGTCCACATTGTTTCTGTCCCGGGCACCTGACAGGCAAACACTGTCAAAGAGAGAAATGCTTTGAACCACAACTGCACAGGTTT
TTCCATGAGAATGATATGTGGTACAGGTTCGAACGCGCCGGGGTGGCTAAATGTAGGTGTAAAGGCCCGGACGCACATTGCAAGC
TTCTTGCTTCACAGGCATGTCCTACGAACAAGTGCCTGAACCAGGGTTCCTGCCTCGTGGCAGAGGGTCACCATTTGTGCCATTGC
CGACCTCCGTATACGGGACCTTTTTGCGACTTGGACACGCAGGCTAATTGTTACAAGGATCGAGGTTTGTCATACAGGGGACTTGC
CCGCACGACATTGAGCGGTGCGTCCTGCCAGAGCTGGGCCAGCGAAGCAACCTATAGAAACATGACCGCCGAGCAGGCTCTGTCA
AGAGGGTTGGGTGACCATGCATTCTGCCGGAATCCAGATAACGACATCCGACCTTGGTGTTTTGTGCTCTCCGGAGATAGGTTGTC
ATGGGAGTATTGTGACCTGGCGCCGTGCCAAGTTCCTACACAAGCAACACCTCCGGTTCAAGTGCTGCCGGAAAGACAGAACCTG
CCCCTCCCGCCTCCCTCAGCGGTGCAAAAACCTCAGCCAACAACTCAACCCCCACCTCGGGCGCCCACCCCAGCGAGTCCAGAGGT
CAGTCAACTGCAGATTCCACTTAGTAGGGCTGATTCACTCTCCTGCGGGCAGAGATTGCGGAAGCGCTTGTCATCACTCAGCAGGG
TCGTTGGCGGTTTGGTAGCCCTCCCCGGCGCGCATCCGTATATGGCCGCACTTTACTGGGGGCATAACTTTTGCGCGGGCAGCCTG
ATTGACAGCTGCTGGGTTTTGACAGCAGCCCATTGCCTGCAAGACAGACCGGCACCAGAAGAGCTTACGGTGGTCTTGGGACAGA
GCCGACATAATCAATCCTGTGAACAATGCCAGACGCTGGCGGTACGATCATATCGGCTCCATGAAGCCTTCTCCCCCAATTCATAC
CAACACGATCTCGCACTGCTTAGACTTCGGGAAAGTGCAGACGGGTCCTGTGCGTTGCTTAGCCCTTATGTGCAACCCGTCTGTTT
GCCTTCTGGGGCGGCCCAAACCCCTGAATCTAAAACCGCACTTTGCGAAGTCGCCGGATGGGGTCATCAGTACGAAGGAGCTGAG
GAATACTCCAGTTTTTTGCAGGAAGCTCAGGTCCCATTCATCCCCCTCGAACGCTGCTCAGCGCCTGATGTACATGGCGCAGCGAT
CTTGCCTGGGATGCTTTGCGCCGGCTTTCTGGAGGGAGGCACAGACGCATGTCAGGGTGACAGTGGCGGTCCCCTTGTTTGTGAAG
AGGGCACGGCGGAATTGCGCCTTACGCTCCGGGGTATAATCTCCTGGGGAAGCGGGTGTGGCGACAGATATAAACCTGGCGTCTA
CACTAACGTGGCAAACTATTTGGCTTGGATACAAGAACATACGGCATCAGGCAGTGGCTCCCACCATCATCACCACCACTAAGCTT

Pig FXII

ATGAGGGCCTGGATCTTCTTTCTCCTTTGCCTGGCCGGGAGGGCTCTGGCAGCTAGCATCCCGCCGTGGAAGGATCCAAGGAAGCA
TAAAGTTATGGCGAGTGAGCATACAGTTGTTCTGACAGTCACTGGGGAACCTTGCCATTTCCCATTCCAATATTACCGCCAGTTGT
ACTACAAATGTATCCAGCGAGGACAACGCGGGCCTCGACCGTGGTGTGCAACGACTCCCAACTTCGAGAAGGACCAACGATGGGC
CTACTGTCTGGAACCCATGAAGGTAAAGGACCACTGTAACAAAGGGAATCCATGCCAAAAAGGGGGTACTTGCGTGAATATGCCC
AACGGACCACACTGCATCTGTCCCGACCATTTTACTGGCAAGCACTGTCAAAAAGAAAAGTGTTTTGAACCTCAGTTTTTGCAGTT
CTTCCAAGAAAACGAGATTTGGCATCGCTTCGAGCCTGCAGGAGTGTCAAAGTGTCAGTGTAAAGGACCAAAAGCTCAGTGCAAA
CCTGTGGCATCACAGGTATGCAGCACCAACCCGTGTCTCAACGGTGGATCTTGCCTTCAAACTGAAGGCCACAGACTTTGCCGCTG
TCCGACGGGTTACGCTGGACGCTTGTGTGATGTTGACCTTAAAGAACGATGTTACTCAGATCGGGGACTGTCTTATCGAGGGATGG
CACAAACTACTCTCTCCGGAGCACCGTGCCAGCCGTGGGCGAGTGAGGCTACATATTGGAACATGACCGCAGAACAGGCTCTCAA
TTGGGGACTCGGGGATCATGCCTTCTGCCGCAATCCCGATAACGATACCCGCCCTTGGTGCTTTGTGTGGAGGGGAGATCAACTCA
GTTGGCAGTATTGTAGACTCGCGCGCTGCCAAGCGCCGATCGGAGAAGCGCCGCCAATCTTGACACCCACTCAAAGTCCATCCGA
GCACCAGGACTCCCCGCTCCTGTCTCGAGAGCCGCAACCAACTACACAAACTCCAAGTCAAAATCTGACAAGCGCTTGGTGTGCG
CCACCCGAACAGAGAGGTCCTCTGCCTTCAGCGGGGCTGGTGGGCTGTGGGCAGAGACTTCGCAAAAGACTCAGTTCCCTGAATC
GCATCGTAGGCGGGCTGGTAGCGTTGCCTGGGGCACACCCTTATATAGCGGCCTTGTATTGGGGTCAGAATTTTTGTGCTGGTAGT
TTGATTGCACCATGCTGGGTCCTTACTGCGGCCCACTGCCTTCAGAATAGGCCGGCTCCCGAGGAGTTGACTGTTGTATTGGGTCA
AGATCGACATAATCAGAGCTGTGAACAATGTCAAACCCTGGCCGTGCGGAGCTATAGACTGCATGAGAGTTACAGTCCAAAGACG
TATCAGCATGATCTCGCGCTGGTAAGATTGAAGGAAACAGCCGACGGCTGCTGTGCTCACCCTAGCCCTTTTGTTCAACCGGTCTG
CCTTCCGCGGAGCGTAGCGAGTAGCGCTGAACCCGAAGGAGCTCTCTGTGAGGTTGCGGGTTGGGGGCATCAGTTTGAAGGTGCA
GAAGAATATAGTTCATTTCTCCAAGAAGCACAGGTCCCTCTGATAAGCCCCGAGAGATGTAGTGCCGCTGACGTGCATGGAGCCG
CCTTCACACCTGGCATGCTCTGCGCCGGCTTCCTCGAGGGGGGCACTGACGCATGCCAAGGTGATAGCGGCGGACCCTTGGTCTGT
GAAGATGAGACTGCCGAACGGCAGCTGGTCCTGCGCGGGATCGTGAGCTGGGGGTCCGGCTGTGGTGATAGGTTGAAGCCTGGCG
TGTACACGGATGTGGCGAACTACCTGGCATGGATACAAGAGCACACCACCAGTGGCAGTGGCTCCCACCATCATCACCACCACTA
AGCTT

Inhibition of recombinant FXII from different species

We expressed recombinant rabbit and pig FXII as the zymogen and activated them to FXIIa by incubation with dextran sulfate (Supplementary Fig. 7). As a control for the recombinant expression and zymogen activation procedure, we also cloned human FXII and found that the activated protease had the same activity as activated FXII derived from human blood and was

inhibited by FXII900 with essentially the same K_i (0.51 nM; Fig. 2c). Rabbit FXIIa was inhibited by FXII900 with a similar K_i (0.90 ± 0.04 nM) as human FXIIa, and pig FXIIa with an around 40-fold weaker K_i (17.4 ± 1.4 nM; Fig. 2c). This result explained the lower activity of FXII900 in inhibiting intrinsic coagulation in pig plasma. Measurement of the K_is for the precursor FXII618 showed that the introduced amino acid changes substantially improved the binding to FXIIa of all species, including pig FXIIa that was improved more than 10-fold (Fig. 2c).

Coagulation assays

The coagulation parameters aPTT and PT were determined in blood plasma using a STAGO STart4 Coagulation analyzer (Diagnostica). Citrated plasma from human (single donor), mouse (CD1, Innovative grade), rabbit (NZW, Innovative grade), and pig (Innovative grade) were supplied by Innovative Research. Coagulation was monitored by an electromagnetically induced movement of a steel ball in the plasma. The time until the ball stopped moving was recorded as the coagulation time. Frozen plasma samples were thawed for around 30 minutes at RT before use. For aPTT measurements in human plasma, 100 µl of plasma, containing or not containing inhibitor, was placed into the cuvette, 100 µl of Pathromtin* SL (silicon dioxide particles, plant phospholipids in HEPES buffer system; Siemens) was added and incubated for two minutes at 37°C in the device before the coagulation was triggered by the addition of 100 µl of pre-warmed (37°C) $CaCl_2$ solution (25 mM, Siemens). For aPTT measurements in mouse, rabbit, and pig plasma, 100 µl of plasma was placed into the cuvette, 100 µl of Dade Actin (Cephalin, Ellagic acid in HEPES buffer system; Siemens) was added and incubated for three minutes at 37°C in the device before coagulation was triggered by addition of 100 µl of pre-warmed (37°C) $CaCl_2$ solution. For PT measurements, 50 µl of human, mouse, rabbit or pig plasma, containing or not containing inhibitor, was placed into the cuvette and incubated for one minute at 37°C before coagulation was trigged by the addition of 100 µl of pre-warmed (37°C) Innovin (recombinant human tissue factor, synthetic phospholipids, and calcium in stabilized HEPES buffer system; Dade Behring/Siemens).

Supplementary References

1. Madhusudhan, M. S. *et al.* Comparative Protein Structure Modeling. in *The Proteomics Protocols Handbook* 831–860 (Humana Press, 2005). doi:10.1385/1-59259-890-0:831

2. Baeriswyl, V. *et al.* A Synthetic Factor XIIa Inhibitor Blocks Selectively Intrinsic Coagulation Initiation. *ACS Chem. Biol.* **10**, 1861–1870 (2015).
