# Supplementary material for: Cyclic peptide FXII inhibitor provides safe anticoagulation in a thrombosis model and in artificial lungs
Source: Nat Commun. 2020 Aug 4;11:3890. doi: 10.1038/s41467-020-17648-w (PMC7403315; doi:10.1038/s41467-020-17648-w)
Supplement: Supplementary file 3 — Reporting Summary [file 41467_2020_17648_MOESM3_ESM.pdf]

nature research

Corresponding author(s): Christian Heinis

Last updated by author(s): May 29, 2020

Reporting Summary

Nature Research wishes to improve the reproducibility of the work that we publish. This form provides structure for consistency and transparency in reporting. For further information on Nature Research policies, see Authors & Referees and the Editorial Policy Checklist .

Statistics

For all statistical analyses, confirm that the following items are present in the figure legend, table legend, main text, or Methods section.

n/a	Confirmed	
☐	☒	The exact sample size (*n*) for each experimental group/condition, given as a discrete number and unit of measurement
☐	☒	A statement on whether measurements were taken from distinct samples or whether the same sample was measured repeatedly
☐	☒	The statistical test(s) used AND whether they are one- or two-sided<br>*Only common tests should be described solely by name; describe more complex techniques in the Methods section.*
☒	☐	A description of all covariates tested
☒	☐	A description of any assumptions or corrections, such as tests of normality and adjustment for multiple comparisons
☐	☒	A full description of the statistical parameters including central tendency (e.g. means) or other basic estimates (e.g. regression coefficient) AND variation (e.g. standard deviation) or associated estimates of uncertainty (e.g. confidence intervals)
☐	☒	For null hypothesis testing, the test statistic (e.g. *F*, *t*, *r*) with confidence intervals, effect sizes, degrees of freedom and *P* value noted<br>*Give P values as exact values whenever suitable.*
☒	☐	For Bayesian analysis, information on the choice of priors and Markov chain Monte Carlo settings
☒	☐	For hierarchical and complex designs, identification of the appropriate level for tests and full reporting of outcomes
☒	☐	Estimates of effect sizes (e.g. Cohen's *d*, Pearson's *r*), indicating how they were calculated

Our web collection on statistics for biologists contains articles on many of the points above.

Software and code

Policy information about availability of computer code

Data collection	LabSolutions (version 5) for LC-MS data collection, i-Control (version 1.1) for data collection with fluorescence plate reader
Data analysis	GraphPad Prism (version 5), Excel (version 2016), ImageJ (version 1.52)

For manuscripts utilizing custom algorithms or software that are central to the research but not yet described in published literature, software must be made available to editors/reviewers. We strongly encourage code deposition in a community repository (e.g. GitHub). See the Nature Research guidelines for submitting code & software for further information.

Data

Policy information about availability of data

All manuscripts must include a data availability statement. This statement should provide the following information, where applicable:

- Accession codes, unique identifiers, or web links for publicly available datasets
- A list of figures that have associated raw data
- A description of any restrictions on data availability

The data supporting the findings of this study are available within the article and Supplementary Information. The source data underlying Figures 1a, 1c–e, 2a–c and 3a–d and Supplementary Figures 7 and 8 are provided as a Source Data file.

Field-specific reporting

Please select the one below that is the best fit for your research. If you are not sure, read the appropriate sections before making your selection.

☒ Life sciences ☐ Behavioural & social sciences ☐ Ecological, evolutionary & environmental sciences

Life sciences study design

All studies must disclose on these points even when the disclosure is negative.

Sample size	Pharmacokinetics in mice: three animals for each time point, different animals for different time points (reason for sample size: minimal number to derive SD).<br>Pharmacokinetics in rabbits: three (iv) and four (sc) animals per group (reason for sample size: minimal number to derive SD).<br>Pharmacokinetics in pigs: three animals per group (reason for sample size: minimal number to derive SD).<br>Mouse thrombosis model: eight to ten animals per group (as indicated) (reason for sample size: arbitrary based on previous experience and variability found for this animal model; the sample size was found to be suited to reach sufficiently small SD values).<br>Mouse bleeding study: six animals per group, four additional animals for heparin group (reason for sample size: arbitrary based on previous experience and variability found for this animal model; the sample size was found to be suited to reach sufficiently small SD values; for the heparin group, we repeated the experiment with four additional mice because the variability for the blood loss was large).<br>ECMO study in rabbits: four animals in the FXII900 group, three animals in the PBS group, two animals in the heparin group reason for sample size: arbitrary based on previous experience and variability found for this animal model; the sample size was found to be suited to reach sufficiently small SD values for comparing the FXII900 and PBS groups).
Data exclusions	Mice showing no speckled pattern at the site of $FeCl_3$ application were assumed to be not injured at the blood vessel and were excluded from the analysis, as described in the methods section. The exclusion criteria was defined after the establishment of the method in the lab using untreated mice.
Replication	FXIIa inhibition assays (independent measurements): FXII618 (3), FXII700 (3), FXII800 (3), FXII850 (2), FXII851 (2), FXII900 (5)<br>Specificity profiling (independent measurements): FXII (5), plasmin, APC (3), all other proteases (2).<br>Stability measurements (independent measurements): FXII850, FXII900 (2), FXII618 (4).<br>In vitro coagulation assay aPTT (independent measurements): FXII618/human plasma (2), FXII900/human plasma (2), FXII618/mouse plasma (2), FXII900/mouse plasma (2), FXII900/rabbit plasma (2), FXII900/pig plasma (2).<br>In vitro coagulation assay PT (independent measurements): FXII618/human plasma (2), FXII900/human plasma (2), FXII900/mouse plasma (2), FXII900/rabbit plasma (3), FXII900/pig plasma (3).<br>Inhibition of recombinant FXIIa from different species (independent measurements): human rFXII (2), mouse rFXII (2), rabbit rFXII (3), pig rFXII (3).<br>Rabbit plasma sample (iv) analysis (independent measurements): concentration (2 or 3), aPTT (2 or 3).<br>Rabbit plasma sample (sc) analysis (independent measurements): concentration (2), aPTT (2).<br>Pig plasma sample (iv) analysis (independent measurements): concentration (2).<br>Analysis of recombinantly expressed FXII orthologs by SDS-PAGE: repetition of the analysis gave similar results .<br>All attempts at replication were successful.
Randomization	Mice and rabbits awere randomly assigned to groups. Pigs were all in one group (no randomization). Human plasma from a single donor was divided into equal aliquots (no randomization).
Blinding	No blinding was applied because the readout was performed by instruments (peptide quantification by LC-MS, aPTT by coagulation analyzer, thrombosis by microscope and camera, etc.)

Reporting for specific materials, systems and methods

We require information from authors about some types of materials, experimental systems and methods used in many studies. Here, indicate whether each material, system or method listed is relevant to your study. If you are not sure if a list item applies to your research, read the appropriate section before selecting a response.

Materials & experimental systems

n/a	Involved in the study	
☒	☐	Antibodies
☐	☒	Eukaryotic cell lines
☒	☐	Palaeontology
☐	☒	Animals and other organisms
☒	☐	Human research participants
☒	☐	Clinical data

Methods

n/a	Involved in the study	
☒	☐	ChIP-seq
☒	☐	Flow cytometry
☒	☐	MRI-based neuroimaging

Eukaryotic cell lines

Policy information about cell lines

Cell line source(s)	CHO cells were obtained from Thermo Fisher Scientific.
Authentication	The cell line used was not authenticated in the laboratory.
Mycoplasma contamination	Cells were not tested for mycoplasma contamination.
Commonly misidentified lines (See ICLAC register)	No commonly misidentified cell lines were used.

Animals and other organisms

Policy information about studies involving animals; ARRIVE guidelines recommended for reporting animal research

Laboratory animals	Male C57BL/6J wild-type mice, 8-20 weeks old<br>Female New Zealand white rabbits, 10-20 weeks old<br>Swiss large white pigs, both sexes, 3-4 months old
Wild animals	None
Field-collected samples	None
Ethics oversight	All experiments in mice and pigs were conducted in accordance with the terms of the Swiss animal protection law and were approved by the animal experimentation committee of the cantonal veterinary service (Canton of Berne, Switzerland). The pharmacokinetic studies in rabbits were performed at Washington Biotech Inc. following ethical standards for animal studies of the Office for Laboratory Animal Welfare (OLAW), a division of the US Public Health Service as administered by the National Institutes for Health. The extracorporeal circulation studies in rabbits were performed in compliance with the Allegheny Health Network Institutional Animal Care and Use Committee. The studies in rabbits were approved by the American Association for Laboratory Animal Science (IACUC).

Note that full information on the approval of the study protocol must also be provided in the manuscript.
